# Fingerprinting Breast Milk; insights into Milk Exosomics

**DOI:** 10.14806/ej.29.0.1048

**Published:** 2024-05-22

**Authors:** Eleni Papakonstantinou, Konstantina Dragoumani, Antonia Mataragka, Flora Bacopoulou, Christos Yapijakis, Nikolaos AA Balatsos, Katerina Pissaridi, Dimitris Ladikos, Aspasia Eftymiadou, George Katsaros, Evangelos Gikas, Pantelis Hatzis, Martina Samiotaki, Michalis Aivaliotis, Vasileios Megalooikonomou, Antonis Giannakakis, Costas Iliopoulos, Erik Bongcam-Rudloff, Sofia Kossida, Elias Eliopoulos, George P Chrousos, Dimitrios Vlachakis

**Affiliations:** 1 Laboratory of Genetics, Department of Biotechnology, School of Applied Biology and Biotechnology, Agricultural University of Athens, Athens, Greece; 2 University Research Institute of Maternal and Child Health & Precision Medicine, and UNESCO Chair on Adolescent Health Care, National and Kapodistrian University of Athens, “Aghia Sophia” Children’s Hospital, Athens, Greece; 3 Department of Biochemistry and Biotechnology, University of Thessaly, Biopolis, Larissa, Greece; 4 YIOTIS S.A., Athens, Greece; 5 Institute of Technology of Agricultural Products, Hellenic Agricultural Organization “DEMETER”, Lykovrisi, Greece; 6 Laboratory of Analytical Chemistry, Department of Chemistry, National and Kapodistrian University of Athens, Athens, Greece; 7 Institute for Fundamental Biomedical Research, Biomedical Sciences Research Center Alexander Fleming, Athens, Greece; 8 Institute for Bioinnovation, Biomedical Sciences Research Center “Alexander Fleming“, Vari, Greece; 9 Basic and Translational Research Unit, Special Unit for Biomedical Research and Education, School of Medicine, Aristotle University of Thessaloniki, Thessaloniki, Greece; 10 Multidimensional Data Analysis and Knowledge Management Laboratory, Computer Engineering and Informatics Department, School of Engineering, University of Patras, Patras, Greece; 11 Laboratory of Gene Expression, Molecular Diagnostics and Modern Therapeutics, Department of Molecular Biology and Genetics, Democritus University of Thrace, Alexandroupolis, Greece; 12 School of Informatics, Faculty of Natural & Mathematical Sciences, King’s College London, London, U.K.; 13 Department of Animal Biosciences, Swedish University of Agricultural Sciences, Uppsala, Sweden; 14 IMGT^®^, the international ImMunoGenetics information system^®^, Laboratoire d’ImmunoGénétique Moléculaire LIGM, Institut de Génétique Humaine, (IGH), Centre National de la Recherche Scientifique (CNRS), Université de Montpellier (UM), Montpellier, France

## Abstract

Breast milk, often referred to as “liquid gold,” is a complex biofluid that provides essential nutrients, immune factors, and developmental cues for newborns. Recent advancements in the field of exosome research have shed light on the critical role of exosomes in breast milk. Exosomes are nanosized vesicles that carry bioactive molecules, including proteins, lipids, nucleic acids, and miRNAs. These tiny messengers play a vital role in intercellular communication and are now being recognized as key players in infant health and development. This paper explores the emerging field of milk exosomics, emphasizing the potential of exosome fingerprinting to uncover valuable insights into the composition and function of breast milk. By deciphering the exosomal cargo, we can gain a deeper understanding of how breast milk influences neonatal health and may even pave the way for personalized nutrition strategies.

## Introduction

Breastfeeding has long been recognized as the “gold standard” of infant nutrition, providing not only essential nutrients but also numerous bioactive molecules crucial for a newborn’s growth and development. While the composition of breast milk has been extensively studied, recent research has unveiled a new layer of complexity - milk exosomes. Exosomes, small extracellular vesicles (EVs) measuring around 50–150 nanometers, are now known to be present in breast milk and are believed to play a pivotal role in infant health (Melnik *et al*., 2021). Herein, we will delve into the field of milk exosomics, exploring what exosomes are, their potential significance in breast milk, and how fingerprinting these exosomes can provide valuable insights into milk composition and its effects on infant health.

## Exosomes: Tiny Messengers with Big Potential

Exosomes constitute a subset of EVs secreted by various cell types, including epithelial cells, immune cells, and even mammary epithelial cells. These vesicles are enclosed by a lipid bilayer membrane and carry a cargo of proteins, lipids, nucleic acids, and microRNAs (miRNAs) from their parent cells (Mosquera-Heredia *et al*., 2021). Exosomes serve as messengers between cells, facilitating intercellular communication and the transfer of information (Kalluri and LeBleu, 2020).

Exosome composition is highly dynamic and dependent on the originating cell type (Mathieu *et al*., 2019). Common components of exosomes include proteins, lipids, nucleic acids, and other biomolecules, for example, exosomes contain a diverse array of proteins, including those involved in membrane transport and fusion (*e.g*., tetraspanins), heat shock proteins, cytoskeletal proteins, and enzymes (Zhang *et al*., 2019). Exosomal membranes are rich in cholesterol, sphingomyelin, and phosphatidylserine, contributing to exosome stability and uptake by target cells (Mosquera-Heredia *et al*., 2021; Zhang *et al*., 2019). Exosomes carry various forms of nucleic acids, including DNA, mRNA, and miRNA, which can be functional when transferred to recipient cells, influencing gene expression. Exosomes have diverse functions in physiological and pathological processes, modulating immune responses, promoting tissue repair, and contributing to cell signaling and homeostasis (Zhang *et al*., 2019). Importantly, exosomes are implicated in various diseases, including cancer, neurodegenerative disorders, and inflammatory conditions (He *et al*., 2023; Kogure *et al*., 2013; Song *et al*., 2015).

## Exosomes in Breast Milk

Breast milk stands as a quintessential source of nutrition for infants, offering a tailored blend of nutrients, growth factors, immunoglobulins, and cells essential for their growth and immune development(Lessen and Kavanagh, 2015). Recent studies have unveiled the presence of a significant quantity of exosomes in breast milk, introducing a novel dimension to our understanding of its role in infant health (Galley and Besner, 2020).

These exosomes, characterized by their lipid bilayer membrane, encapsulate a cargo of bioactive molecules, including immune-related proteins and microRNAs (miRNAs), which hold promise in modulating various physiological processes in infants. The cargo of exosomes in breast milk is of particular interest. Exosomes in breast milk carry immune-related proteins and miRNAs, potentially aiding in the development of the infant’s immune system.

Proteomic analysis of breast milk-derived exosomes has unveiled a diverse repertoire of proteins intricately involved in various cellular components, molecular functions, and biological processes, including immune-related processes such as defense response, phagocytosis, complement activation, and regulation of cytokine responses. Notably, these exosomes carry growth factors crucial for tissue development, such as the gastrointestinal tract (Hock *et al*., 2017; Pisano *et al*., 2020). Studies have illuminated the multifaceted role of breast milk EVs in modulating both innate and adaptive immune responses. They stimulate specific Toll-like receptors (TLRs) while concurrently tempering the response of endosomal TLRs, suggesting a regulatory role in immune activation (Kim *et al*., 2023). Additionally, milk EVs have been shown to inhibit the activation and differentiation of CD4+ T cells, implying their involvement in regulating adaptive immune responses and facilitating immune maturation in infants (Zonneveld *et al*., 2021).

Proteomic profiling of milk EVs across different mammal species and lactation stages has unraveled a diverse array of proteins with significant nutritional, enzymatic, hormonal, and immunomodulatory functions, and these proteins underpin the various properties of milk EVs, including their immunomodulatory effects and potential as biomarkers for assessing mammary gland health. Notably, the protein cargo of EVs exhibits dynamic changes during lactation stages, with colostrum EVs enriched in immune-related proteins and mature milk EVs harboring proteins involved in ribosome regulation and cell growth. Understanding the protein content of milk EVs provides valuable insights into milk’s nutritional value and its potential applications in health assessment (Buratta *et al*., 2023).

The lipid composition of milk EVs constitutes a pivotal determinant of their properties and biological effects. Enriched in sphingolipids and glycerophospholipids containing saturated fatty acids, EV membranes exhibit rigidity and stability in biological fluids (Tenchov *et al*., 2022). These lipids also serve as precursors for bioactive molecules involved in immune signaling and inflammation. Despite abundant research on the nucleic acid cargo of milk EVs, information regarding their lipid content and biological roles remains limited. Initial investigations have revealed a shared lipid composition enriched in phosphatidylserine (PS) and sphingomyelin (SM) among milk EVs from bovine and human sources compared to milk fat globules (MFG). These findings underscore the resemblance of milk EVs to those isolated from other body fluids and cell culture media. Recent studies have delved into characterizing the lipid profiles of milk EVs, unveiling high levels of phosphatidylcholine (PC) and phosphatidylethanolamine (PE) in bovine milk EVs, with PS enrichment compared to milk. Similarly, human milk EVs exhibit diverse lipid species, with discernible differences between preterm and term samples. Notably, these lipid variances may contribute to the prevention of conditions like necrotizing enterocolitis (NEC) through the modulation of signaling pathways (Buratta *et al*., 2023).

Metabolomic analysis of human milk (HM) involves the identification and annotation of metabolites utilizing various databases and spectral libraries. Techniques such as NMR, LC-MS, CE-MS, and GC-MS facilitate the detection of a comprehensive array of metabolites in HM, including carbohydrates, fatty acids, amino acids, and organic acids. Common metabolites detected across multiple analytical platforms include glucose, lactose, amino acids like tyrosine, fatty acids such as capric acid/caprate and caprylic acid/caprylate, and organic acids like citric acid/citrate and pyruvic acid/pyruvate. These metabolites represent vital sources of energy, essential for infant growth and development, emphasizing the nutritional richness of human milk (Ramos-Garcia *et al*., 2023). The lipidomic and metabolomic profile of milk EVs have the potential to unveil their diverse biological functions and potential therapeutic applications. Understanding these components provides valuable insights into the nutritional value of milk and its implications for infant health and development.

Breast milk exosomes also carry small RNAs, such as mRNAs, lncRNAs, siRNAs and more, which can influence gene expression and regulation in the infant (Melnik *et al*., 2021). MicroRNAs (miRNAs) found in breast milk play a crucial role in regulating various physiological processes in infants. The selective packaging of miRNAs into exosomes suggests a regulated process rather than random selection, highlighting the potential significance of exosomes in early neonatal development. The composition of miRNAs in human breast milk varies depending on factors such as gestational age at delivery and lactation stage, with preterm milk exhibiting distinct miRNA profiles compared to term milk. Changes in miRNA expression patterns in preterm milk may reflect adaptations to promote neurodevelopment and mitigate the challenges faced by preterm infants, contributing to the maturation of the preterm infant’s brain (Slyk-Gulewska *et al*., 2023).

Furthermore, specific miRNAs detected in human breast milk target genes involved in synaptic components, neurogenesis, and neurodevelopmental processes, suggesting their potential roles in regulating neuronal function and connectivity. Dysregulation of these miRNAs has been implicated in various neurological disorders, highlighting their importance in maintaining proper brain function and development. Future research directions may include longitudinal studies to further investigate the evolution of miRNA cargo in human breast milk over lactation stages and its potential impact on infant neurodevelopment. Additionally, miRNA sequencing of the whole milk sample could provide insights into differences in miRNA composition between exosomal and non-exosomal fractions (Freiria-Martinez *et al*., 2023).

Studies have examined the influence of different factors on the miRNA profile of breast milk and its implications for infant health. Research indicates that while the total concentration of miRNAs remains relatively stable during the first 6 months of lactation, significant changes occur in miRNA composition, particularly at 4 months compared to 2 months postpartum, suggesting an adaptation of miRNA profiles to meet the evolving needs of the infant. Additionally, mothers who deliver prematurely exhibit altered hormonal profiles and miRNA expression in their breast milk, potentially benefiting premature infants by influencing glucose homeostasis, adipogenesis, and immune function (Slyk-Gulewska *et al*., 2023).

The delivery method also impacts miRNA expression in breast milk, with cesarean delivery disrupting the balance of specific miRNAs and potentially increasing the risk of type 2 diabetes mellitus (T2DM) later in life (Slyk-Gulewska *et al*., 2023). Furthermore, correlations between maternal stress levels and changes in extracellular vesicle-derived miRNAs suggest potential impacts on fatty acid metabolism, steroid biosynthesis, and organ growth regulation in infants. Breast milk from overweight or obese mothers contains altered levels of miRNAs associated with infant growth and neurodevelopment, possibly contributing to an increased risk of childhood obesity due to lower levels of certain miRNAs. Regarding diabetes, breast milk from mothers with gestational diabetes mellitus (GDM) exhibits abnormal miRNA levels associated with metabolic outcomes in their infants. Similarly, mothers with type 1 diabetes mellitus (T1DM) demonstrate differences in miRNA composition in breast milk exosomes, potentially impacting immune response processes. Breastfeeding protects against T2DM development, partly due to the absence of certain miRNAs present in cow’s milk, which may contribute to β-cell dysfunction and insulin resistance. Breastfeeding also facilitates epigenetic programming through exosome miRNAs, whereas infant formulas lack these bioactive components, potentially modulating gene expression related to obesity predisposition and reducing the risk of obesity and related diseases. Understanding the diverse roles of miRNAs in breast milk and their modulation by various factors holds promise for the prevention and treatment of various diseases in infants, including necrotizing enterocolitis (NEC), atopic diseases, diabetes, obesity, and cancer. Further research is imperative to elucidate the specific mechanisms and therapeutic potential of miRNAs in breast milk (Slyk-Gulewska *et al*., 2023).

Human milk exosomes also contain various miRNAs associated with adipogenesis, the process of fat cell development, while concerns have arisen regarding the potential transfer of adipogenic miRNAs across species through milk consumption, particularly as humans consume milk from other animals throughout their lives (Abbas *et al*., 2023). However, evidence linking dairy milk consumption to increased obesity rates remains inconclusive. Effective biological action of milk exosomal miRNAs relies on sufficient quantities reaching target cells. Studies have found that the miRNA profiles in the milk of obese women differ, with lower levels of miRNA-148a and miRNA-30b observed. This variation may influence fat accumulation in breastfeeding infants. Certain milk exosomal miRNAs, such as miRNA-29, miRNA-148, miRNA-30b, and miRNA-125b, are believed to have epigenetic effects on recipients, potentially impacting gene expression and cellular function. Furthermore, milk exosomes possess the ability to traverse the gastrointestinal barrier, making them promising candidates for oral drug delivery. They can also be engineered and loaded with specific miRNAs involved in adipocyte differentiation, conversion, or browning, offering potential therapeutic benefits. By modifying the miRNA cargo of exosomes, it may be possible to enhance their health-promoting effects and provide an alternative to traditional pharmaceutical interventions (Abbas *et al*., 2023).

Additional research has investigated the therapeutic potential of human milk exosomes in treating necrotizing enterocolitis (NEC) using a rat model, where both exosomes from full-term and preterm human breast milk were found to alleviate NEC severity, with preterm-derived exosomes showing better promotion of intestinal epithelial cell proliferation (Yan *et al*., 2022). High-throughput sequencing revealed differential expression of long non-coding RNAs (lncRNAs) and messenger RNAs (mRNAs) between the exosome types, with associated pathways implicated in cell proliferation, such as such as the JAK-STAT signaling pathway, bile secretion, and the AMPK signaling pathway, highlighting the protective role of human milk exosomes against NEC and giving insight into potential therapeutic mechanisms (Yan *et al*., 2022).

While the exact functions of breast milk exosomes are still being elucidated, several potential roles have been proposed. Exosomes may help educate the infant’s immune system by delivering immune-related molecules and miRNAs. Growth factors carried by exosomes may contribute to the development and maturation of the infant’s gastrointestinal tract and other tissues. Exosomes may play a role in shaping the infant’s gut microbiota, which has a significant impact on overall health (Slyk-Gulewska *et al*., 2023).

## Fingerprinting Breast Milk Exosomes

Fingerprinting breast milk exosomes involves the comprehensive analysis of their cargo to understand their composition and potential effects on infant health. This process combines techniques from various scientific disciplines, including proteomics, genomics, metabolomics, lipidomics, and miRNA profiling (Shenker *et al*., 2020). Proteomic analysis of breast milk exosomes involves the identification and quantification of the proteins they carry. Mass spectrometry-based approaches are commonly used to determine the protein composition, providing insights into the functional roles of exosomes in breast milk (Beck *et al*., 2015). Genomic and miRNA analysis involves sequencing the nucleic acids carried by breast milk exosomes. This can reveal the specific miRNAs and genes involved in immune modulation, tissue development, and other processes crucial for infant health (Hunt *et al*., 2011). Lipidomic and metabolomic analysis focuses on the lipid content and metabolite cargo of exosomes, respectively. This can uncover lipid signatures associated with breast milk exosomes, shedding light on their stability and potential interactions with recipient cells (Garwolinska *et al*., 2017).

Fingerprinting breast milk exosomes has the potential to have significant implications for infant health and nutrition. By deciphering the exosomal cargo in breast milk, it may be possible to tailor breastfeeding advice and strategies to meet the specific needs of individual infants (Shenker *et al*., 2020). Personalized nutrition based on exosomal profiles could optimize the health and development of newborns. Breast milk exosomes may serve as biomarkers for various maternal and infant health conditions (Mourtzi *et al*., 2021). Changes in exosomal cargo could indicate infections, metabolic disorders, or other health issues in either the mother or the infant. Understanding the cargo of breast milk exosomes could lead to the development of novel therapeutic approaches. Exosomes could be isolated and used as targeted drug delivery vehicles, or their cargo could inspire the development of new therapies for infant health issues.

While the field of milk exosomics holds great promise, it also faces several challenges and areas for further research. Standardisation of exosome isolation and analysis methods is essential for reproducibility and comparability of research findings. Understanding the functional significance of breast milk exosomes and their cargo remains a priority (Mitsis *et al*., 2020). In vitro and in vivo studies are needed to validate their roles in infant health, as well as longitudinal studies required to assess the long-term effects of breast milk exosomes on infant health and development. A holistic omics profiling of human milk exosomes, whilst further bioinformatics and AI analysis is in hand, will be able to advance our knowledge on their specific role and the personalized benefits for the afterbirth biological interplay between the mother and the infant (Papakonstantinou *et al*., 2023). As exosome research advances, ethical considerations regarding the collection and use of breast milk samples for research purposes need to be addressed.

## Conclusion

Breast milk exosomes represent a fascinating frontier in the field of neonatal nutrition and health. These tiny vesicles, carrying a cargo of proteins, lipids, and nucleic acids, have the potential to shape the development and well-being of newborns. Fingerprinting breast milk exosomes through proteomic, genomic, and lipidomic analysis can provide invaluable insights into milk composition and its impact on infant health. As research in milk exosomics continues to advance, we may unlock the secrets of personalized nutrition for infants and discover new therapeutic avenues for addressing neonatal health challenges. While many questions remain unanswered, the future of milk exosomics holds great promise for improving the health and well-being of the youngest members of our society. Further research in this domain promises to uncover novel strategies for enhancing infant nutrition and mitigating health risks.

## Key Points

- Exosomes in breast milk exhibit diverse cargo including proteins, metabolites, and nucleic acids, crucial for intercellular communication and immune modulation. Understanding their dynamic composition and roles in physiological processes provides insights into their significance in infant health and development.
- Breast milk exosomes carry immune-related proteins and microRNAs, contributing to the infant’s immune system development. They stimulate specific immune receptors while regulating immune responses, suggesting a regulatory role in immune activation and maturation, thus highlighting their potential in enhancing infant immunity.
- The composition of microRNAs in breast milk exosomes varies with factors like gestational age and delivery method, potentially influencing neurodevelopment in infants, whilst dysregulation of these microRNAs has been linked to neurological disorders, underscoring the importance of breast milk exosomes in promoting proper brain function and development.
- Milk exosomes show promise in treating conditions like necrotizing enterocolitis (NEC) and as potential biomarkers for maternal and infant health conditions. They could be harnessed for targeted drug delivery or inspire the development of novel therapies, indicating their therapeutic potential in neonatal health.
- While milk exosome research offers exciting possibilities, standardisation of isolation methods and understanding their functional significance remain critical. Longitudinal studies are needed to assess their long-term effects on infant health, and ethical considerations regarding sample collection and use must be addressed as the field advances.
